# Acute-phase serum amyloid A for early detection of hepatocellular carcinoma in cirrhotic patients with low AFP level

**DOI:** 10.1038/s41598-022-09713-9

**Published:** 2022-04-06

**Authors:** Jin-Lin Wu, Tung-Hung Su, Pei-Jer Chen, Yun-Ru Chen

**Affiliations:** 1 Ph.D. Program for Cancer Biology and Drug Discovery, China Medical University and Academia Sinica, Taichung, Taiwan; 2 Genomics Research Center, Academia Sinica, Taipei, Taiwan; 3 Division of Gastroenterology and Hepatology, Department of Internal Medicine, National Taiwan University Hospital, Taipei, Taiwan; 4 Hepatitis Research Center, National Taiwan University Hospital, Taipei, Taiwan; 5 Graduate Institute of Clinical Medicine, National Taiwan University College of Medicine, Taipei, Taiwan; 6 Department of Medical Research, National Taiwan University Hospital, Taipei, Taiwan

**Keywords:** Hepatocellular carcinoma, Prions, Protein aggregation

## Abstract

Regular hepatocellular carcinoma (HCC) surveillance by ultrasonography in combination with the α-fetoprotein (AFP) examination is unsatisfactory in diagnostic sensitivity for early-stage HCC especially in cirrhotic patients. We conducted a prospective study in a tertiary medical center in Taiwan and consecutively collected serum samples from patients with chronic hepatitis, liver cirrhosis (LC), or HCC for new biomarker discovery. Overall, 166 patients were enrolled, including 40 hepatitis, 30 LC, and 96 HCC. Four acute-phase serum amyloid A (A-SAA) derived biomarkers including total A-SAA, A-SAA monomer and oligomer, and protein misfolding cyclic amplification (PMCA) signal were measured and compared between patients with and without HCC. A-SAA biomarkers significantly increased in the HCC group when compared to the hepatitis and LC groups, and generally increased in more advanced tumor stages. Among A-SAA biomarkers, the area under the receiver operator characteristic curves (AUROCs) for PMCA signal in discrimination of all-stage and early-stage HCC were 0.86 and 0.9 in cirrhotic patients, which is comparable to AFP. For cirrhotic patients with low AFP (< 7 ng/mL), PMCA signal maintained good capacity in prediction of early-stage HCC (AUROC: 0.94). Serum A-SAA and its prion-like property showed a potential to complement AFP in detection of early-stage HCC.

## Introduction

Hepatocellular carcinoma (HCC) is the most common primary liver cancer and the third leading cause of cancer-related death worldwide^1^. The incidence of HCC depends on variable prevalence of chronic hepatitis B virus (HBV) and hepatitis C virus (HCV) infection^2^. Liver cirrhosis developed after prolonged hepatic injuries caused by HBV or HCV infections, alcoholic hepatitis, nonalcoholic steatohepatitis, or autoimmune hepatitis^3^. Cirrhosis is the major risk for HCC, and approximately 80% to 90% of HCC patients already have established cirrhosis^4^. The Barcelona Clinic Liver Cancer (BCLC) system classifies HCC into five stages based on the number, size, and extension of tumors, liver reserve, and the overall performance status of the patients^5^. Currently, curative therapies are only feasible for very early and early-stage (BCLC stage 0-A) HCC^6^, suggesting the importance of early HCC detection. At present, the clinical guidelines of European Association for the Study of the Liver (EASL)^6^ and American Association for the Study of Liver Diseases (AASLD)^7^ recommend the high-risk patients to receive abdominal ultrasound imaging every six months with or without the measurement of the α-fetoprotein (AFP) level. However, ultrasonography is limited by its image quality and operator-dependent^8^. Serum AFP level remains normal in over 30% HCC patients^9^. The sensitivity of ultrasonography for HCC detection with or without consideration of the AFP level was 63% or 45%, respectively, which was unsatisfactory^10^. There is an urgent need to develop new biomarkers for early HCC detection.

Serum amyloid A (SAA) is a family of positive acute-phase reactants produced by hepatocytes^11^. Among four family members, SAA1 and SAA2, also referred to as “acute-phase SAA” (A-SAA), share high sequence identity^12^ and the expression increases significantly during an acute-phase response^13^. As an amyloid protein, A-SAA is prone to aggregate and form insoluble fibrils with cross-β structures and these fibrils can be probed by an amyloid dye thioflavin T (ThT)^14^. Furthermore, formation of amyloid fibrils is inducible and can be amplified by a method called protein misfolding cyclic amplification (PMCA)^15^. The serum SAA level increases in several malignancies including lung^16^, pancreas^17^, colon^18^, kidney^19,20^, and stomach^21^. SAA has also been detected in HCC cell lines and tissues^22–24^. The serum SAA level positively correlates with tumor stages^25,26^. Serum SAA can be used to monitor the recurrence of nasopharyngeal cancer and breast cancer after treatments^27,28^ and to predict the overall survival^29^. Serum SAA intensifies in HBV-related HCC^30^, and may serve as a prognostic marker for HCC^31^. These results suggest SAA is a pan-cancer marker; however, the contribution of A-SAA among the four SAAs was not specified. In addition, the role of A-SAA in detection of early-stage HCC remains unclear.

Here, we aimed to evaluate the potential of A-SAA as a biomarker for HCC. We first measured and analyzed the concentration of total serum A-SAA in patients with hepatitis, liver cirrhosis, and HCC. Then we specifically examined A-SAA monomer and oligomer of the three patient groups. Since SAA is an amyloidogenic protein, by using the amyloidogenic property we further investigated the capacity of a method namely protein misfolding cyclic amplification (PMCA), with prion-like amplification of A-SAA to discriminate HCC from non-HCC groups. PMCA is a method to allow amplification of the signal in body fluids^15^. Finally, we evaluated the predictability of A-SAA in patients with low AFP level and found PMCA signal maintained good capacity in prediction of early-stage HCC.

## Results

### Total A-SAA concentration is significantly higher in HCC patients than in hepatitis and LC patients

**Figure 1:**
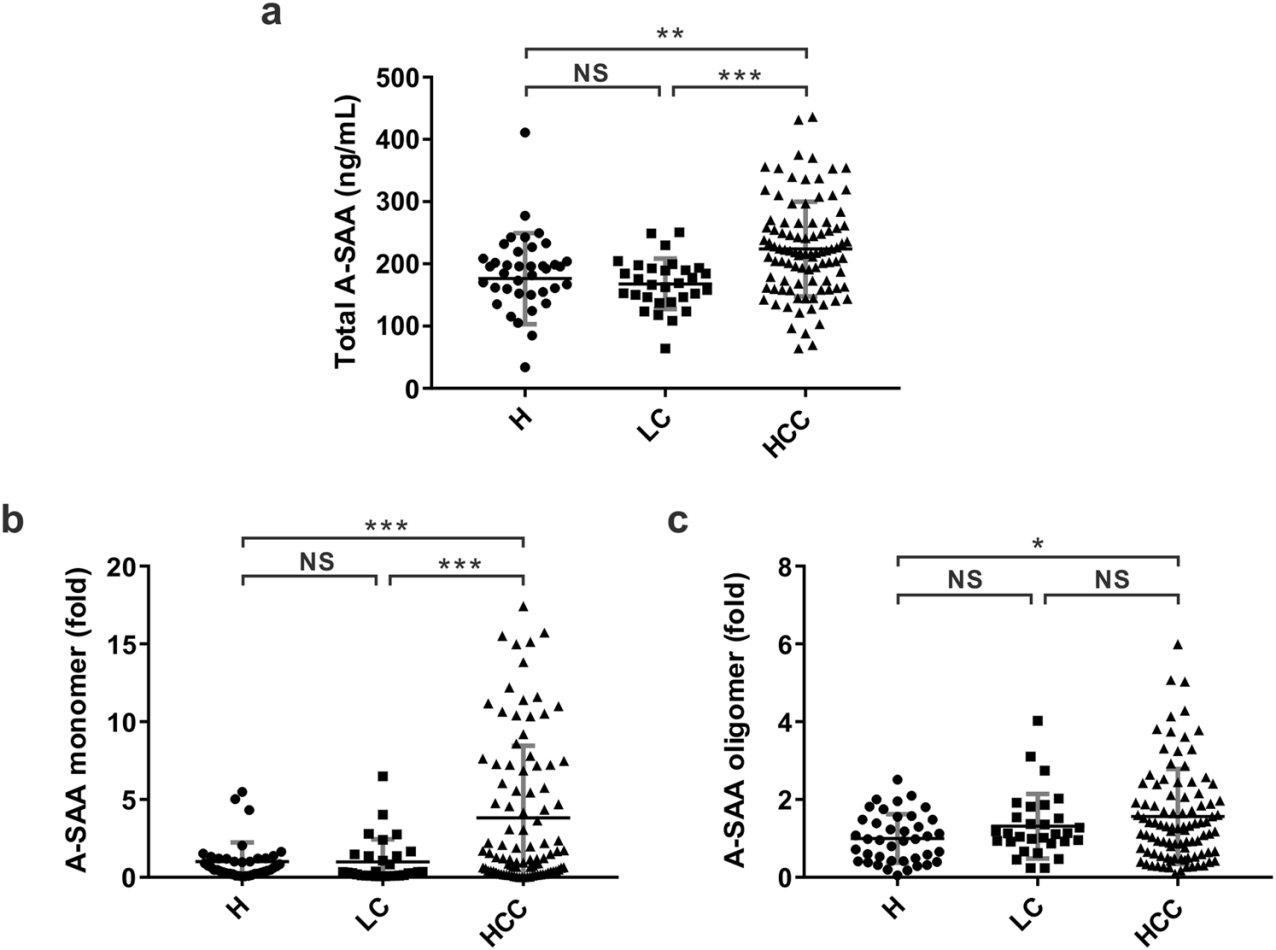
Comparison of A-SAA derived biomarker levels among hepatitis, LC, and HCC patients. A total of 166 serum samples from 40 hepatitis (indicated as H), 30 LC, and 96 HCC patients were measured and analyzed. (**a**) Total A-SAA. (**b**) A-SAA monomer. (**c**) A-SAA oligomer. The statistical analysis was performed by one-way ANOVA and Tukey’s Post Hoc Test, where *P* < 0.05 (*),  < 0.01 (**), and < 0.001 (***). *NS* not significant.

To assess the potential of A-SAA as an HCC biomarker, a total of 166 patients were prospectively enrolled, and they were categorized into three groups: hepatitis (n = 40), LC (n = 30), and HCC (n = 96). Their demographic and clinical characteristics are listed in Table 1. The mean age was 47 in the hepatitis group, compared to 60 in patients of the LC or the HCC groups. There were 43%, 63%, and 72% of male in the hepatitis, LC, and HCC groups, respectively. Regarding the etiology, more than 63% of patients in individual groups had HBV infection. Among the HCC group, 86 patients (90%) had cirrhosis, and 42 patients (44%) had early-stage HCC (BCLC 0 and A). We first employed ELISA for measurement of total A-SAA concentration in the serum collected from the three patient groups. Among the three groups, total A-SAA concentration in the HCC patients (mean ± standard deviation: 223.8 ± 76.0 ng/mL) was significantly higher than in the hepatitis (176.3 ± 73.2 ng/mL, *P* = 0.0013) and LC (167.8 ± 40.6 ng/mL, *P* = 0.0006) patients (Fig. 1a). No significant difference was found in total A-SAA concentration between the hepatitis and LC cohorts.

**Table 1:** Characteristics of study participants.

	HCC (n = 96)	Hepatitis (n = 40)	LC (n = 30)	*P*-value
**Age, mean ± SD, years**	60 ± 11	47 ± 16	60 ± 10	< 0.0001
**Gender, n (%)**				0.0053
Male	69 (72%)	17 (43%)	19 (63%)	
Female	27 (28%)	23 (58%)	11 (37%)	
**Etiology, n (%)**				
HBV	68 (71%)	38 (95%)	19 (63%)	
HCV	27 (28%)	2 (5%)	10 (33%)	
HBV + HCV	0	0	1 (3%)	
Cryptogenic	1 (1%)	0	0	
**Laboratory tests**				
AST (U/L)	80 ± 88	35 ± 41	37 ± 27	0.001
ALT (U/L)	57 ± 51	47 ± 78	35 ± 29	0.1475
**Cirrhosis, n (%)**	86 (90%)	NA	30 (100%)	
**Tumor stage**		NA	NA	NA
BCLC stage 0 (Very early)	11 (11%)			
BCLC stage A (Early)	31 (32%)			
BCLC stage B (Intermediate)	28 (29%)			
BCLC stage C (Advanced)	21 (22%)			
BCLC stage D (Terminal)	5 (5%)			

*ALT* alanine aminotransferase, *AST* aspartate aminotransferase, *BCLC* Barcelona Clinic Liver Cancer, *HBV* hepatitis B virus, *HCC* hepatocellular carcinoma, *HCV* hepatitis C virus, *LC* liver cirrhosis, *NA* not applicable, *SD* standard deviation.

### A-SAA monomer, but not the oligomer, differentiates HCC from LC

Due to the amyloidogenic property of A-SAA, we specifically examined the serum samples in Western blot to observe the possible association between different SAA assemblies and the three patient groups. The serum samples for SDS-PAGE were prepared without the addition of reducing agent β-mercaptoethanol and boiling to preserve oligomeric state of A-SAA. The Western blot results showed two main assemblies of A-SAA proteins in patients’ sera, A-SAA monomer migrated at ~ 9 kDa and its high-molecular-weight oligomer at ~ 130 kDa (Supplementary Fig. S1a). Since monomeric A-SAAs in patients’ sera displayed a size smaller than the theoretic molecular mass of SAA1 (11.7 kDa) and SAA2 (11.6 kDa), we compared the sera along with our purified recombinant full-length human SAA1 in Western blot (Supplementary Fig. S2). The result showed that the serum A-SAA migrated in similar distance to the recombinant SAA1 confirming that serum A-SAA detected is a full-length monomer. The faster migration in SDS-PAGE may be due to protein conformation and/or hydrophobicity.

After Western blot, we quantified the amount of A-SAA monomer and oligomer for further analysis. Since aggregation is a concentration-dependent event, we wondered if the presence of oligomers was due to the increase of A-SAA concentration in serum. Therefore, we conducted a correlation analysis on A-SAA monomer and oligomer. A Spearman’s coefficient of 0.296 (*P* = 0.0001) indicated only a modest positive correlation between A-SAA monomer and oligomer (Supplementary Fig. S1b). Next, we compared the level of both monomer and oligomer of A-SAA among the three groups. The level of A-SAA monomer was significantly higher in the HCC group (3.8 ± 4.6 fold) when compared to that in the hepatitis (1 ± 1.2 fold, *P* = 0.0002) and the LC (1 ± 1.5 fold, *P* = 0.0008) groups (Fig. 1b). No significant difference was found in A-SAA monomer level between the hepatitis and LC groups. The result is consistent with the ELISA result. For A-SAA oligomer, the HCC group (1.6 ± 1.2 fold) had a significantly higher level compared to the hepatitis group (1 ± 0.6 fold, *P* = 0.0125) but not to the LC group (1.3 ± 0.8 fold, *P* = 0.4486) (Fig. 1c).

### PMCA of SAA1 showed potential to differentiate HCC from LC

**Figure 2:**
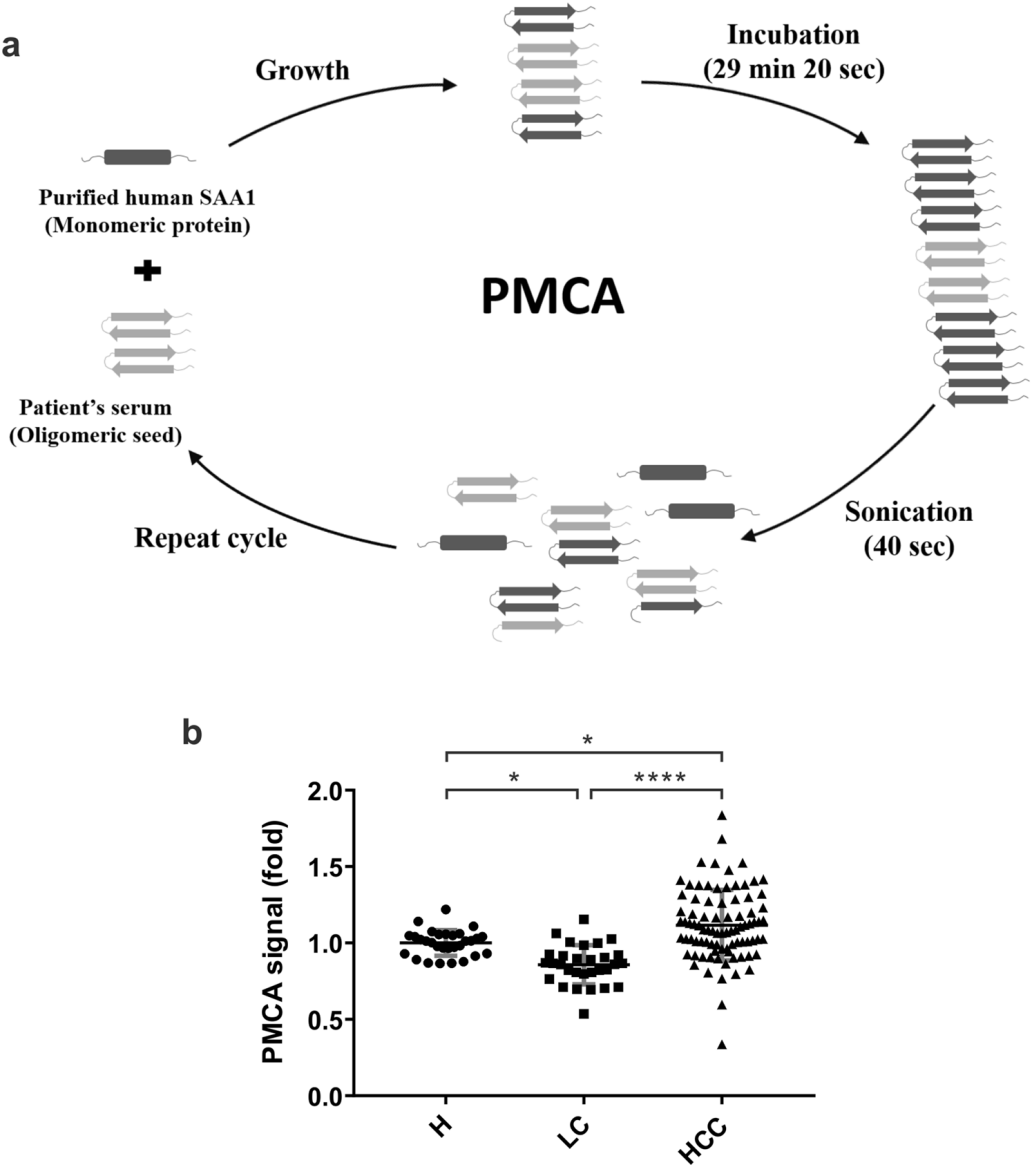
Comparison of PMCA signals among hepatitis, LC, and HCC patients. (**a**) Diagram illustrating the principle of PMCA technique. A standard PMCA procedure starts with incubation of oligomeric seeds with monomeric amyloid protein to allow growth of the polymers. The aggregates are then fragmented by sonication to increase the number of seeds for further aggregate growth. After repeating PMCA cycles, the concentration of initial preformed oligomeric protein can be amplified and thus easy to be detected. (**b**) Comparison of PMCA signals among hepatitis, LC, and HCC patients. A total of 146 serum samples from 30 hepatitis (indicated as H), 30 LC, and 86 HCC patients were measured and analyzed. The statistical analysis was performed by one-way ANOVA and Tukey’s Post Hoc Test, where *P* < 0.05 (*) and < 0.0001 (****). *NS* not significant.

PMCA is a method that utilizes nucleation-dependent amyloid aggregation process to accelerate the conversion of monomeric amyloid protein into fibrils^15^. The principle of PMCA relies on the application of ultrasound waves to fragment the preformed amyloid fibrils, increasing the amount of misfolded seeds present in the samples containing normal monomeric protein and further induce the amplification of aggregation or accelerate the aggregation reaction (Fig. 2a). PMCA has been applied to detect variant Creutzfeldt-Jakob disease and demonstrated a diagnostic capacity with high sensitivity and specificity^15,32^. Recently, PMCA has also been applied to differentiate α-synuclein in Parkinson’s disease and multiple system atrophy^33^. Since SAA also possesses amyloidogenic property, we performed PMCA on SAA and examined the potential of PMCA as detection method for HCC. Our purified recombinant full-length human SAA1 was used as a substrate and serum from each patient as seeds for PMCA. The patient’s serum was mixed with purified SAA1 in a volume ratio of 1:20 in a solution containing amyloid dye ThT that recognized amyloid fibrils. The samples underwent 24 PMCA cycles in which each cycle comprised 29 min 20 s of incubation followed by a 40-s sonication pulse. The final ThT fluorescence was detected and normalized to the signal in hepatitis group. Our results showed that patients with HCC had an increased PMCA signal (1.1 ± 0.2 fold) in comparison with the hepatitis (1 ± 0.1 fold, *P* = 0.0129) and LC (0.9 ± 0.1 fold, *P* < 0.0001) patients (Fig. 2b). Meanwhile, we investigated the relationship of these A-SAA biomarkers and clinical characteristics. The A-SAA biomarkers were not associated with age, sex, HBV or HCV infection, except a significantly higher PMCA signal in HBV carriers (*P* < 0.05) (Supplementary Fig. S3). There was low to moderate correlation between AST/ALT and the total A-SAA or A-SAA monomer levels, but the A-SAA oligomer, or the PMCA signal did not correlate with ALT level, indicating A-SAA might be associated with liver inflammation but the correlation was low. The A-SAA biomarkers did not correlate with liver fibrosis measured by the Fib-4 index. (Supplementary Fig. S4).

### Correlation between A-SAA derived biomarkers and HCC stages

**Figure 3:**
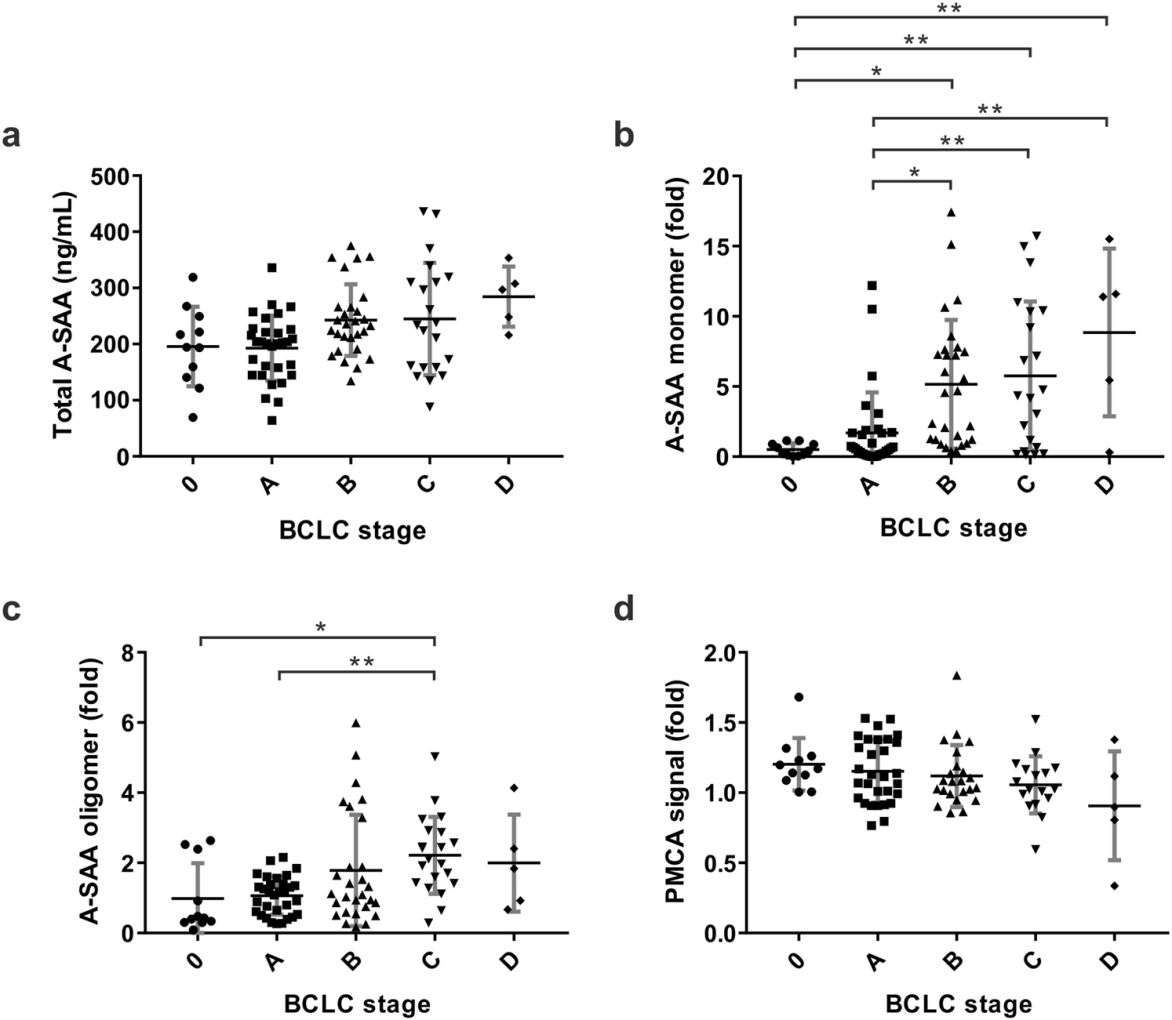
The level of A-SAA derived biomarkers at different HCC BCLC stages. A total of 96 serum samples from stage 0 (n = 11), stage A (n = 31), stage B (n = 28), stage C (n = 21), and stage D (n = 5) were measured and analyzed. (**a**) Total A-SAA. (**b**) A-SAA monomer. (**c**) A-SAA oligomer. (**d**) PMCA signal. The statistical analysis was performed by one-way ANOVA and Tukey’s Post Hoc Test, where *P* < 0.05 (*) and < 0.01 (**).

The level of A-SAA biomarkers including total A-SAA, A-SAA monomer, A-SAA oligomer, and A-SAA PMCA signal was further compared within HCC patients. The mean concentration of total A-SAA for HCC patients at BCLC stage 0, A, B, C, and D were 195.7 ± 70.9, 192.7 ± 57.9, 242.7 ± 63.9, 244.8 ± 99.8, and 284.5 ± 53.5 ng/mL, respectively. No significant difference between any two BCLC stages was detected (Fig. 3a). The mean level of A-SAA monomer increased significantly in BCLC stage B (5.2 ± 4.6 fold), stage C (5.8 ± 5.3 fold), and stage D (8.8 ± 6.0 fold) when compared to BCLC stage 0 (0.5 ± 0.4 fold, *P* = 0.0163, 0.0075, and 0.0025), or BCLC stage A (1.7 ± 2.9 fold, *P* = 0.0139, 0.0061, and 0.0042) (Fig. 3b). The mean level of A-SAA oligomer also significantly increased in BCLC stage C (2.2 ± 1.1 fold) when compared to BCLC stage 0 (1.0 ± 1.0 fold, *P* = 0.0366) and stage A (1.1 ± 0.5 fold, *P* = 0.0052) (Fig. 3c). The PMCA signals were comparable at different BCLC stages without significance detected between any two groups (Fig. 3d). Overall, A-SAA monomer and oligomer showed an increased level in more advanced stage HCC in comparison with early-stage HCC.

### The prediction of all-stage and early-stage HCC in all patients using A-SAA derived biomarkers

**Figure 4:**
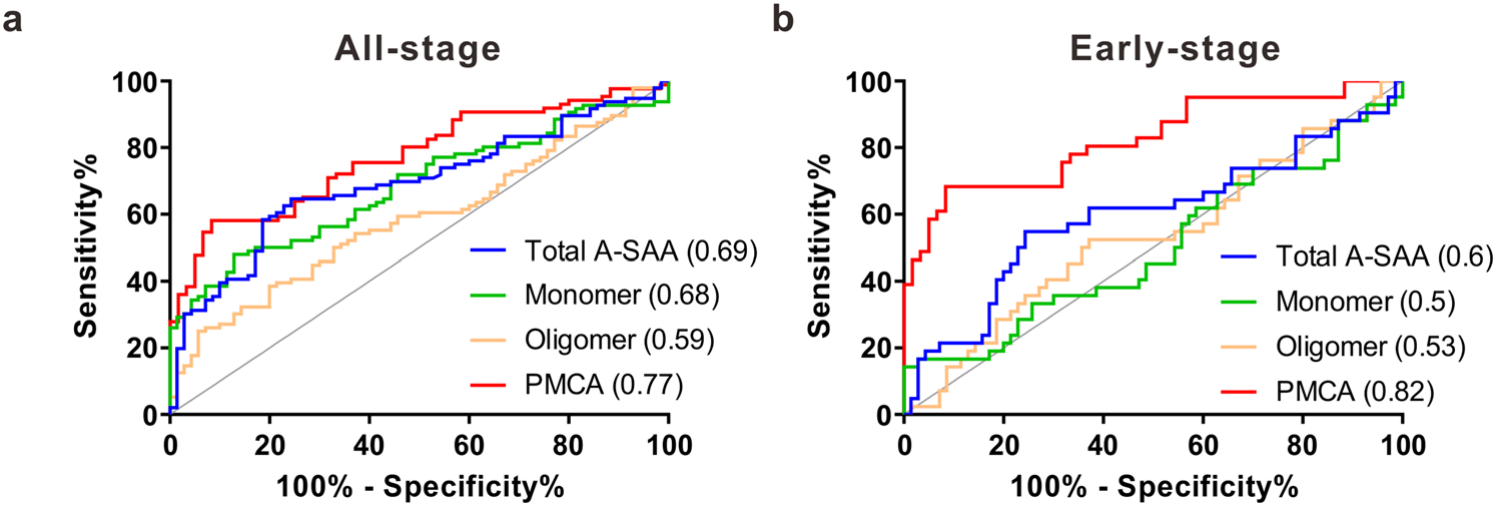
ROC curves of A-SAA derived biomarkers in differentiating all-stage and early-stage HCC in all patients. (**a**) Detection of all-stage HCC. (**b**) Detection of early-stage HCC. AUROC value for each curve is shown in parentheses.

Receiver Operating Characteristic (ROC) analysis was performed to assess the prediction value of A-SAA derived biomarkers in discriminating all-stage and early-stage HCC in all patients (Fig. 4). As a comparison, among all the biomarkers, AFP showed the highest area under the ROC curve (AUROC) of 0.9 (95% CI 0.84–0.95, *P* < 0.0001) to distinguish all-stage HCC (Supplementary Table S1). The best A-SAA biomarker was PMCA signal which demonstrated an AUROC of 0.77 (95% CI 0.7–0.85, *P* < 0.0001) (Fig. 4a and Supplementary Table S1). With the optimal cut-off values, AFP showed a higher sensitivity of 78% compared to 58% for PMCA signal with similar specificities of 93% and 92%, respectively.

To further investigate the performance of A-SAA biomarkers in detecting early-stage HCC, we only included 42 early-stage HCC (BCLC 0-A) patients along with hepatitis and LC patients in this subgroup analysis. Among all the biomarkers, the highest AUROC was 0.89 (95% CI 0.81–0.96, *P* < 0.0001) for AFP followed by PMCA signal (0.82; 95% CI 0.74–0.91, *P* < 0.0001) (Fig. 4b and Supplementary Table S1). The sensitivity of AFP was 74% compared to 68% of PMCA signal with specificity of 93% and 92%, respectively. To summarize, for all patients, AFP demonstrated the best performance in detection of all-stage HCC whereas PMCA signal showed a comparable capacity in early-stage HCC prediction.

### The prediction of HCC using A-SAA derived biomarkers among cirrhotic patients

**Figure 5:**
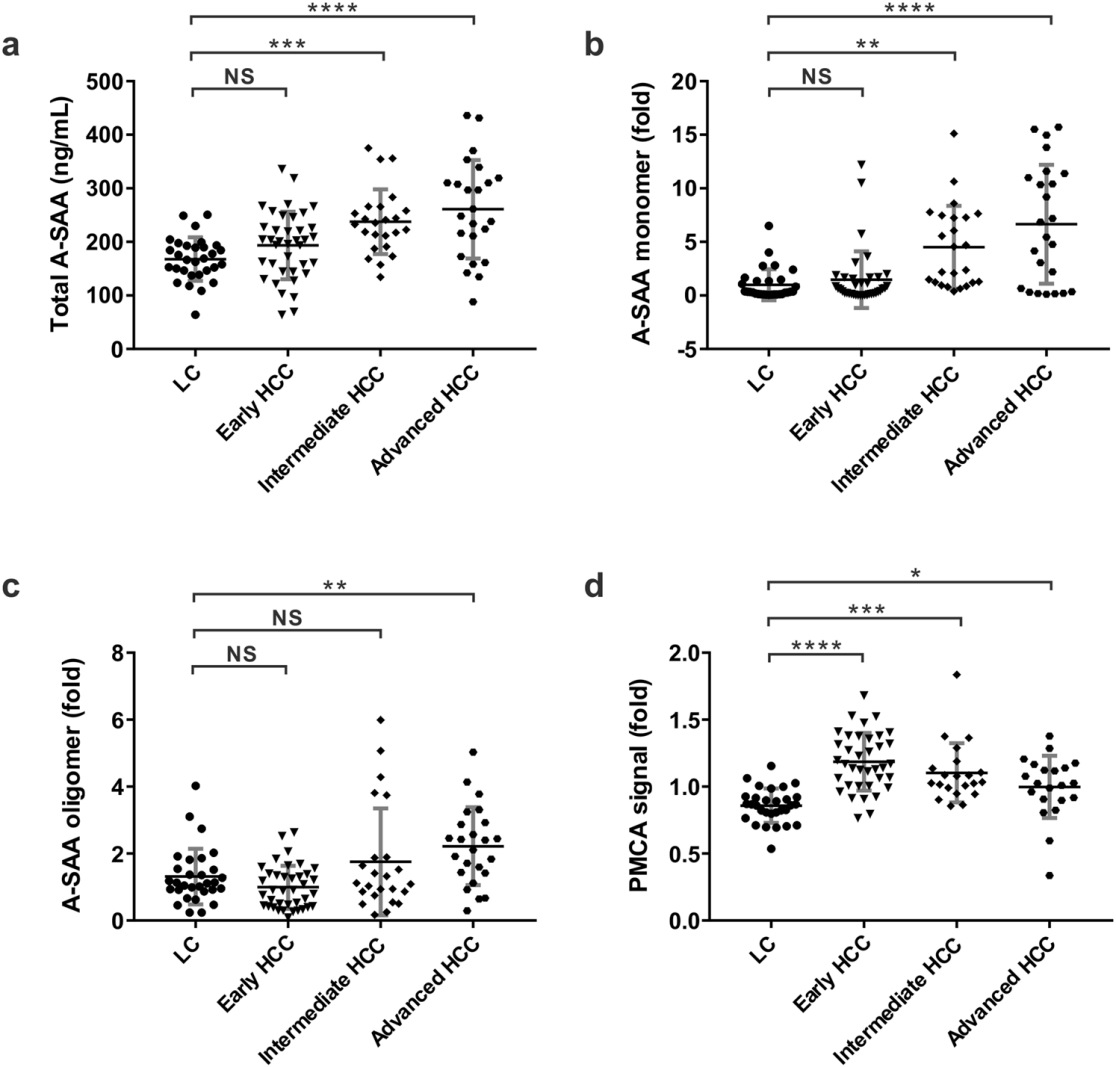
Comparison of A-SAA derived biomarkers in patients with cirrhosis. A total of 116 serum samples from 30 LC and 86 HCC patients were measured and analyzed. Early HCC indicates BCLC stage 0-A (n = 38). Intermediate HCC indicates BCLC stage B (n = 24). Advanced HCC indicates BCLC stage C-D (n = 24). (**a**) Total A-SAA. (**b**) A-SAA monomer. (**c**) A-SAA oligomer. (**d**) PMCA signal. The statistical analysis was performed by one-way ANOVA and Tukey’s Post Hoc Test, where *P* < 0.05 (*),  < 0.01 (**), < 0.001 (***), and < 0.0001 (****). *NS* not significant.

Since patients with cirrhosis had the highest risk of HCC, we investigated the HCC predictability of A-SAA biomarkers among cirrhotic patients. From 166 patients, we excluded 50 non-cirrhotic patients and only included 116 cirrhotic patients in this subgroup analysis. We found a significantly lower concentration of total A-SAA in the LC group when compared to the intermediate (BCLC B, *P* = 0.0005) and the advanced HCC group (BCLC C-D, *P* < 0.0001), but not to the early HCC group (BCLC 0-A, *P* = 0.2569) (Fig. 5a). A-SAA monomer showed a pattern similar to total A-SAA with a significantly lower level in the LC group when compared to the intermediate (*P* = 0.0012) and the advanced HCC groups (*P* < 0.0001), but not to the early HCC group (*P* = 0.9021) (Fig. 5b). For A-SAA oligomer, a significantly lower level was found in the LC group only when compared to the advanced HCC group (*P* = 0.0065) (Fig. 5c). PMCA signal of the LC group showed a significantly lower level when compared to the early (*P* < 0.0001), the intermediate (*P* = 0.0001), and the advanced HCC groups (*P* = 0.0399) (Fig. 5d). Together, among the A-SAA derived biomarkers, PMCA signal is the only one able to differentiate LC and the early-stage HCC.

**Figure 6:**
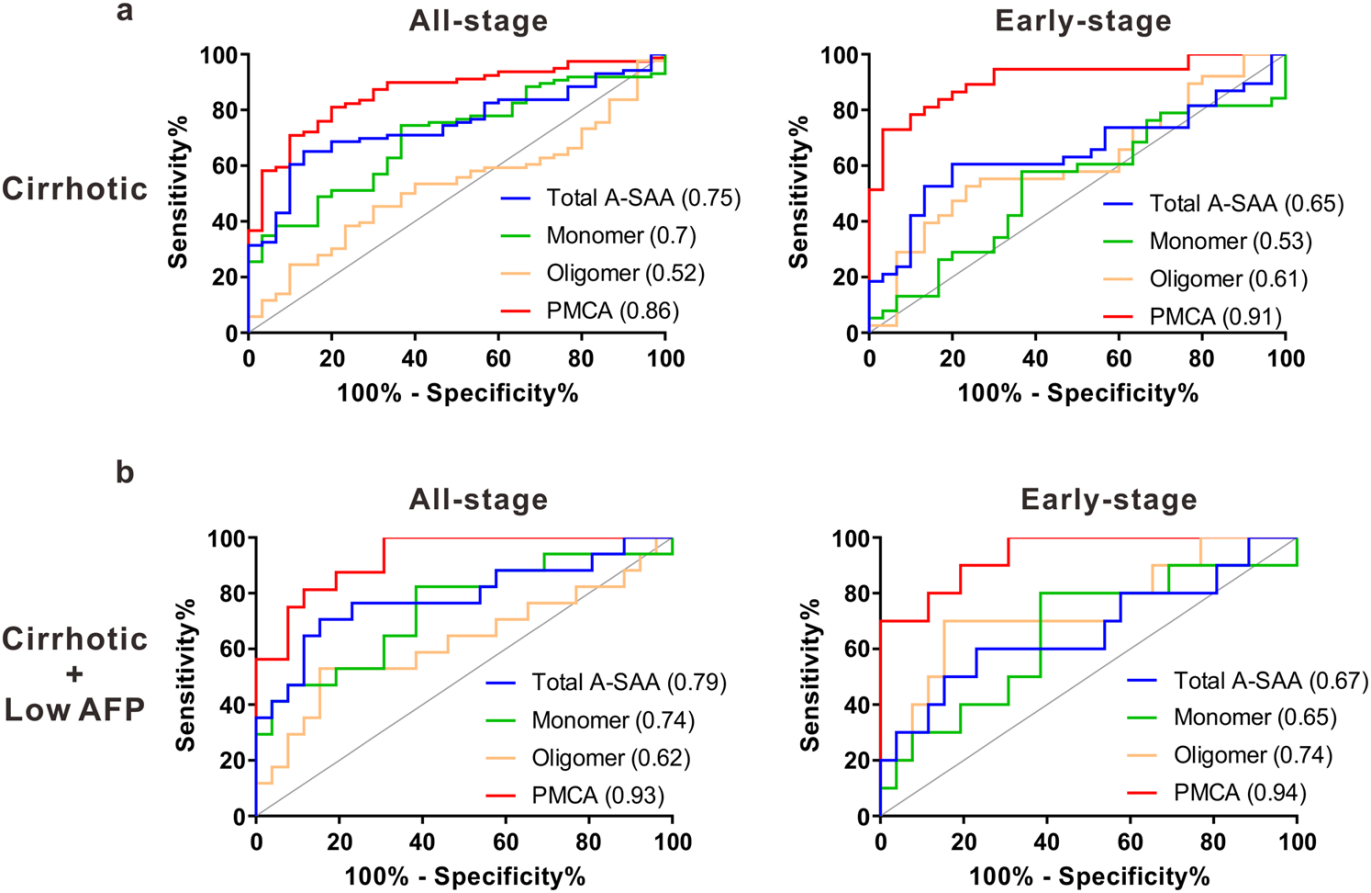
ROC curves of A-SAA derived biomarkers in discrimination of all-stage and early-stage HCC in cirrhotic patients with or without low AFP level. (**a**) Detection of HCC in all cirrhotic patients. (**b**) Detection of early-stage HCC in cirrhotic patients with low AFP. AUROC value for each curve is shown in parentheses.

For detection of all-stage HCC in cirrhotic patients, the highest AUROCs were comparable for PMCA signal (0.86; 95% CI 0.79–0.93, *P* < 0.0001) and AFP (0.84; 95% CI 0.76–0.92, *P* < 0.0001) (Fig. 6a and Supplementary Table S2). The HCC detection sensitivity at the optimal cut-off values was also similar for PMCA signal (81%) and AFP (80%) as well as their specificity (80% vs. 83%, respectively). For early-stage HCC detection, AFP and PMCA signal again had a statistically comparable AUROC of 0.81 (95% CI 0.7–0.93, *P* = 0.0002) and 0.91 (95% CI 0.84–0.98, *P* < 0.0001), respectively (Fig. 6a and Supplementary Table S2). The sensitivities were close for AFP (74%) and PMCA signal (73%) with a higher specificity for PMCA signal (97%) compared to AFP (83%). In patients with cirrhosis, PMCA signal and AFP showed a similar detection capacity for all-stage HCC. For early HCC detection, with their optimal cut-off values, PMCA signal demonstrated a better performance than AFP with a similar sensitivity but a higher specificity.

### Performance of A-SAA derived biomarkers in cirrhotic patients with low AFP level

Because it is difficult to detect HCC in patients with low AFP level, and A-SAA biomarkers, especially PMCA signal, had better HCC predictability in cirrhotic patients, we only included the patients with both cirrhosis and low AFP (< 7 ng/mL, optimal cut-off decided by AUROC) in this subgroup analysis. For all-stage HCC detection, PMCA signal again showed the highest AUROC of 0.93 (95% CI 0.85–1, *P* < 0.0001) followed by total A-SAA (0.79; 95% CI 0.63–0.94, *P* = 0.0017) (Fig. 6b and Supplementary Table S2). The sensitivity and specificity for PMCA signal was 81% and 88%. For early-stage HCC detection, the AUROC of PMCA signal reached 0.94 (95% CI 0.86–1, *P* < 0.0001) with a sensitivity and specificity of 90% and 81% (Fig. 6b and Supplementary Table S2).

Furthermore, we performed multivariable logistic regression analysis to evaluate the association between PMCA signal and HCC after adjusted to age, gender, and AST/ALT concentration (Supplementary Table S3). We separated the PMCA signals for all cases in quartiles (PMCA signal < 0.91 fold [Quartile 1, Q1], 0.91–1.02 fold [Q2], 1.02–1.14 fold [Q3], and ≥ 1.14 fold [Q4]). The adjusted odds ratios for Q2 to Q4 are 8.43 (95% CI 2.01–44.77), 15 (95% CI 3.86–74.36), and 101 (95% CI 19.61–753.6) for all-stage HCC, respectively. After excluding the intermediate and advanced-stage HCC, the PMCA signals were separated in quartiles where the signal < 0.89 [Q1], 0.89–1.00 fold [Q2], 1.00–1.13 fold [Q3], and ≥ 1.13 fold [Q4]. The adjusted odds ratios to predict early-stage HCC for Q2 to Q4 are 17.43 (95% CI 2.32–339.5), 25.96 (95% CI 3.49–512.8), and 344.8 (95% CI 38.47–8286), respectively. After adjustment to confounders, PMCA signal still maintained strong association with all-stage and early-stage HCC.

To conclude, among A-SAA biomarkers, PMCA signal demonstrated the highest HCC detection capacity, especially in the subgroup of cirrhotic patients with low AFP level. Based on our results, we designed a surveillance flow chart to demonstrate HCC detection focused on cirrhotic patients by AFP in combination of PMCA signal (Supplementary Fig. S5). We began with cirrhotic patients including 30 LC and 79 HCC cases. With AFP lower than 7 ng/mL, there are still 16 out of 79 HCC patients (approximately 20% of all HCC patients) remained undetected. In the subgroup of cirrhotic patients, the optimal cut-off value for PMCA signal decided by AUROC was 0.9 fold. In the following detection by PMCA signal with the cut-off, only 1 HCC cases (approximately 1% of all HCC patients) remained undetected.

## Discussion

SAA has been shown to increase in the patients with different cancers and correlate with the progression of tumorigenesis. The majority of studies shows the value of SAA as a biomarker in prognosis of cancers^31,34–36^. In the present study, we attempted to investigate the association between SAA and different stages of liver diseases, especially among three closely related pathogenic conditions: hepatitis, liver cirrhosis, and HCC, and the value of SAA for early HCC detection. From our results, A-SAA derived biomarkers generally showed a higher level in the HCC group compared to the hepatitis and the LC groups. Furthermore, the level of A-SAA monomer increased in more advanced HCC (stage B, C, and D), compared to early HCC (stage 0 and A). The increase in SAA level in more advanced HCC suggests that SAA may not only be an indicator but also be involved in disease progression.

Expression of SAA, especially A-SAA, is induced and regulated by proinflammatory cytokines produced by a variety of immune cells, which are abundant in the tumor microenvironment^37,38^. Moreover, cancer cells also have the capability to produce inflammatory cytokines^39^. SAA has been shown to stimulate the production of matrix metalloproteinases (MMP)^40^ that is positively associated with tumor progression^41,42^. The alteration and degradation of extracellular matrix by MMPs is also closely related to tumor initiation and development^43^. Besides, SAA has been shown to induce tumor cell migration^44^ and angiogenesis^45^, which are involved in cancer exacerbation. Therefore, the significant increase in serum A-SAA of the HCC group is likely to be a feedback loop between SAA and hepatocarcinogenesis. However, the mechanistic insights for the current study are still unclear and required further intensive investigation.

The unique aspect of our study is that we utilized PMCA in combination with the prion-like property of A-SAA as a predictive method for HCC. PMCA method was originally designed to detect amyloidosis-related diseases^46^ and has never been applied and assessed for cancer detection. The basic principle of PMCA relies on an amyloidogenic protein can serve as a seed and induces structural transformation of the native substrate proteins to form ThT dye-recognizable aggregates. From our results, PMCA method showed a better capacity for HCC discrimination compared to total A-SAA measured by ELISA or A-SAA monomer detected by Western blot, especially for early-stage HCC. Since the cancer patients contain more serum A-SAA as evidenced by the level of A-SAA monomer and seeding-liable oligomer, the PMCA procedure may further amplify the signals to enhance differences between the HCC and non-cancer groups. Besides, a more amyloidogenic conformation of A-SAA may be adopted due to the aberrant environment for protein folding or maturation in cancer cells. However, it is possible that the amplification can be induced by other amyloidogenic proteins, but not SAA, in the serum although the hetero-seeding is in general less efficient.

Cirrhotic patients had high risk to develop HCC; however, the low sensitivity of ultrasonography to detect early HCC in cirrhotic background remains a challenge to clinicians. Previous studies have shown that the sensitivity and specificity of AFP for HCC surveillance largely depended on the selected serum level threshold^47^. In our study, even with low threshold of AFP (< 7 ng/mL), approximately 20% of HCC patients still cannot be detected. From our analysis, the PMCA method demonstrated the best HCC predictability in cirrhotic patients with low AFP. The PMCA method may be applied for HCC surveillance in this clinical scenario. However, there are limitations to the present study. In this study, we only included hepatitis, LC, and HCC patients with the background of HBV or HCV infection. Therefore, we do not know the A-SAA level in healthy control, or hepatitis patients with other etiologies, e.g., non-alcoholic fatty liver disease (NAFLD), autoimmune hepatitis (AIH), or alcoholic liver disease. Moreover, since the case numbers are small in this study, further large scale, prospective validation is needed.

In conclusion, by using several methods to investigate HCC predictive capacity of A-SAA, we found that A-SAA derived biomarkers, especially PMCA method, have potential to facilitate HCC detection. Even though currently AFP may still be the main serum biomarker for HCC surveillance, A-SAA biomarkers can be used as a complementary test in combination with imaging examination, especially for the cirrhotic patients with low AFP level.

## Methods

### Serum samples

This was a prospective study conducted at National Taiwan University Hospital (NTUH) since 2012 to investigate the biomarkers for HCC. Patients with chronic liver disease, liver cirrhosis, and HCC (with viable tumor) who were followed regularly in the liver clinic of NTUH were enrolled. Their sera were collected, processed, and stored at -80 °C until further use. Cirrhosis was diagnosed clinically by the nodular liver surface, coarse liver parenchymal texture, narrowed vessels with irregular intrahepatic vessel contour, and enlarged spleen size by abdominal ultrasonography^48^. HCC was diagnosed by either pathology or typical dynamic imaging studies according to the AASLD guideline. The liver function tests and AFP level were measured in the Department of Laboratory Medicine in NTUH. The study conformed to the ethical guidelines of the 1975 Declaration of Helsinki and was approved by the Institutional Review Boards of NTUH (201108073RC and 201207048RIB). All patients provided their written informed consents before enrolment.

### Enzyme-linked immunosorbent assay (ELISA)

The assessment of total A-SAA concentration in the sera from hepatitis, LC, and HCC patients was conducted using Human SAA ELISA kit (ab100635, Abcam, Cambridge, MA, USA). One hundred µL of 20 × PBS-diluted serum sample was added into the antibody-pre-coated ELISA plate and incubated at 4 °C overnight. Next, 100 µL of biotinylated SAA detection antibody was applied to the plate and recognized by addition of 100 µL of horseradish peroxidase-conjugated streptavidin. Chromogenic substrate was added and the absorbance at 450 nm was then measured using SpectraMax M5 microplate reader (Molecular Devices, San Jose, CA, USA).

### Western blot

Proteins in 20 × PBS-diluted serum samples were separated by 8–16% gradient SDS-PAGE precast gels (Bio-Rad, Hercules, CA, USA). To preserve oligomeric states of A-SAA during SDS-PAGE, the sample buffer did not contain β-mercaptoethanol and the samples were not boiled. A-SAA proteins were then recognized by an anti A-SAA antibody (ab687, Abcam) and a secondary antibody (goat anti-mouse IgG peroxidase conjugated antibody, Jackson ImmunoResearch Labs, West Grove, PA, USA) and visualized in a luminescence/fluorescence imaging system ImageQuant LAS 4000 biomolecular imager (GE Healthcare, Marlborough, MA, USA). The amount of A-SAA monomer and oligomer was quantified by ImageQuant TL 8.2 image analysis (GE Healthcare). The final results were calculated by normalization of the amount of A-SAA monomer and oligomer from each patient to the mean amount of the hepatitis group.

### Expression and purification of recombinant human SAA1

Full-length human SAA1 (residues 1–104) was cloned into a pET-14b vector which contains an N-terminal histidine tag (His-tag) and expressed in *Escherichia coli* BL21 cells. A TEV protease cleavage site (ENLYFQS) was added to the N-terminus of mature SAA1 sequence for His-tag removal. Since TEV protease recognizes and cleaves between Q and S, SAA1 after cleavage contains an additional serine residue at the N-terminus. The overexpression of His-tagged SAA1 protein was induced at 37 °C by the addition of 1 mM isopropyl β-D-1-thiogalactopyranoside at mid-exponential growth phase of *E. coli*. After 5-h induction, the bacteria were harvested by centrifugation and lyzed in lysis buffer (20 mM Tris, pH 8.0, 5 M urea, 50 mM imidazole) by a microfluidizer (model M110L, Microfluidics, Westwood, MA, USA). His-tagged SAA1 protein was purified by HisTrap HP column (GE Healthcare) in the presence of 5 M urea. The His-tag was removed from SAA1 by incubation of His-tagged TEV protease in a TEV:SAA molar ratio of 1:20 at 30 °C for 16 h. The cleaved proteins were further purified by HisTrap HP column as the flow-through. The pure SAA1 protein was then dialyzed into a fresh 50 mM phosphate buffer (pH 7.0) and stored at − 80 °C for subsequent experiments.

### Protein misfolding cyclic amplification (PMCA)

For PMCA experiment, the sera of patients and purified recombinant human SAA1 were used as seeds and substrates, respectively. Samples were prepared by mixing patient’s serum with purified SAA1 in a volume ratio of 1:20. As substrate, SAA1 protein was prepared to a final concentration of 20 μM in a 50 mM phosphate buffer (pH 7.0). A final concentration 10 μM of ThT was added into samples to monitor amyloid fibril formation of SAA1. One hundred µL of SAA1-serum sample was loaded into a 96-well ELISA plate and PMCA reaction was automatically performed by a microsonicator (model Q700, Qsonica, Newtown, CT, USA). One PMCA cycle comprised 29 min 20 s of incubation at 25 °C, followed by a 40-s sonication pulse at a potency of 300 to 310 W. A complete PMCA round consisted of 24 cycles that equals to 12 h. The fluorescent emission of ThT was measured at 485 nm using SpectraMax M3 microplate reader (Molecular Devices) with an excitation wavelength of 442 nm. The final results were calculated by normalization of PMCA signal from each patient to the mean signal value of the hepatitis group.

### Statistical analysis

GraphPad Prism version 7.04 and 8.4.2 (GraphPad Software, San Diego, CA, USA) were utilized for statistical analyses. One-way ANOVA was used to evaluate the difference for more than two groups. Post-hoc analyses were conducted by Tukey’s multiple comparison between two groups. Correlation between two variables was measured by the Spearman’s correlation coefficient. Receiver operating characteristic (ROC) curves and area under the ROC curve (AUROC) were generated to assess the predictive capacity of each biomarker, and the Youden index to indicate the optimal threshold for each ROC curve. Multivariable logistic regression was used to evaluate the association of biomarkers with the outcome under adjustment of age, gender, and AST/ALT concentration. A *P*-value (two-tailed) less than 0.05 was considered a statistical significance.

## Supplementary Information

Supplementary Information.

## Data Availability

The datasets used and/or analyzed during the current study available from the corresponding author on reasonable request.
