# Supplementary material for: Acute-phase serum amyloid A for early detection of hepatocellular carcinoma in cirrhotic patients with low AFP level
Source: Sci Rep. 2022 Apr 6;12:5799. doi: 10.1038/s41598-022-09713-9 (PMC8986837; doi:10.1038/s41598-022-09713-9)
Supplement: Supplementary file 1 — Supplementary Information. [file 41598_2022_9713_MOESM1_ESM.pdf]

Supplementary Information

Acute-phase serum amyloid A for early detection of hepatocellular carcinoma in cirrhotic patients with low AFP level

Jin-Lin Wu [1,2], Tung-Hung Su [3,4], Pei-Jer Chen [3,4,5,6], Yun-Ru Chen [1,2*]

[1] Ph.D. Program for Cancer Biology and Drug Discovery, China Medical University and Academia Sinica, Taichung, Taiwan, R.O.C.

[2] Genomics Research Center, Academia Sinica, Taipei, Taiwan

[3] Division of Gastroenterology and Hepatology, Department of Internal Medicine, National Taiwan University Hospital, Taipei, Taiwan

[4] Hepatitis Research Center, National Taiwan University Hospital, Taipei, Taiwan

[5] Graduate Institute of Clinical Medicine, National Taiwan University College of Medicine, Taipei, Taiwan

[6] Department of Medical Research, National Taiwan University Hospital, Taipei, Taiwan

This file includes:

- Figure S1-S5
- Table S1-S3

Figure S1

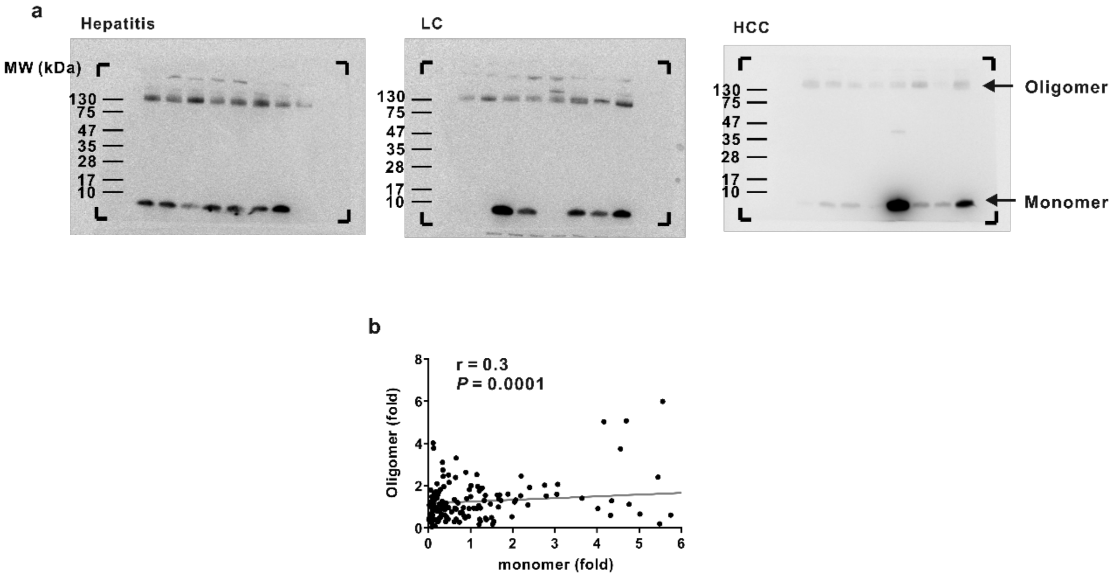

Figure S1. Western blot of A-SAA in sera obtained from hepatitis, LC, and HCC patients. Different assemblies of A-SAA were observed in sera of all three patient groups by western blot without boiling and reducing agent. (**a**) Two main assemblies of A-SAA, A-SAA monomer and high-molecular-weight oligomers formed by A-SAA, were detected. Representative images of the full membrane are shown. The edges of the membranes were marked. (**b**) Correlation between the level of A-SAA monomer and oligomer. The level of correlation was analyzed and evaluated by Spearman's correlation coefficient (indicated as r).

Figure S2

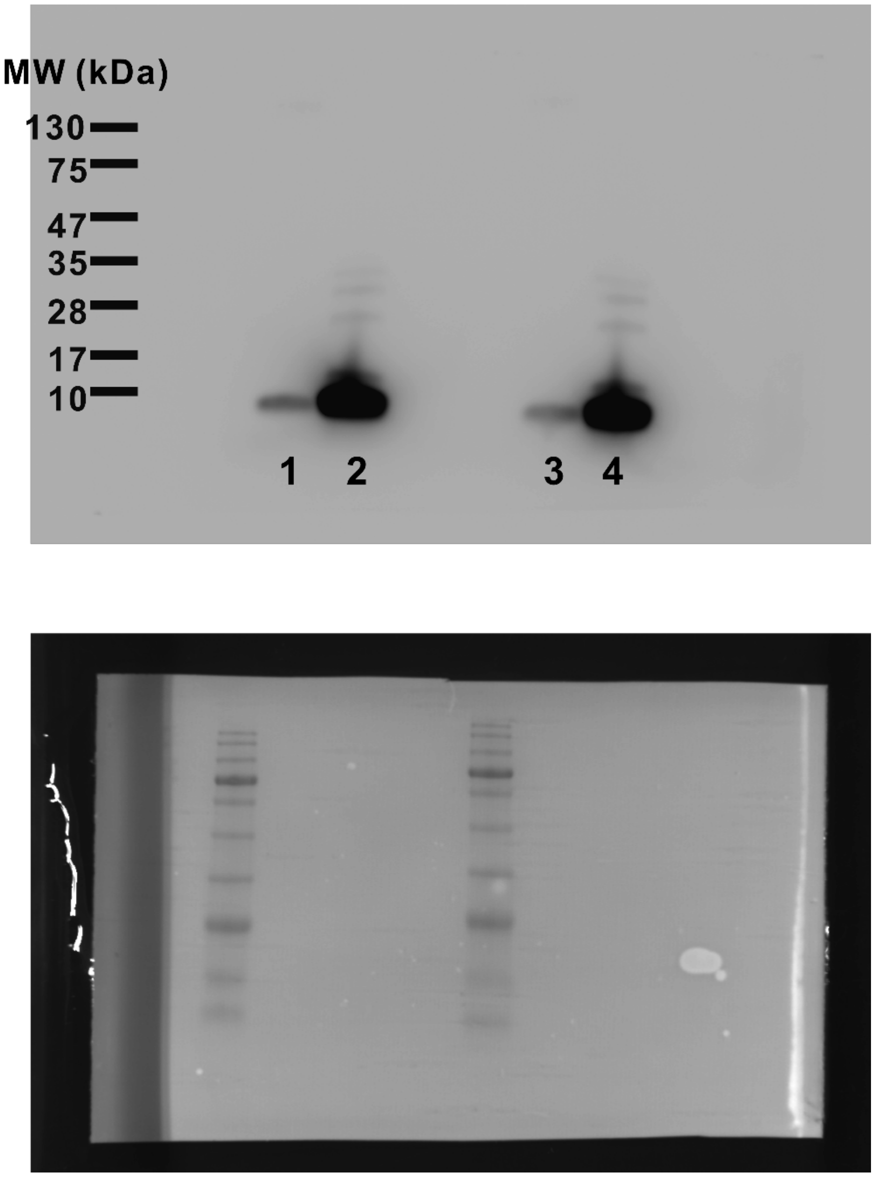

Figure S2. Serum and recombinant full-length A-SAA monomers in SDS-PAGE. A-SAA in patient's serum (lane 1 & 3) and recombinant human SAA1 (lane 2 & 4) were run on SDS-PAGE and subjected to Western blot. Similar migration distance at ~9 kDa was observed for serum A-SAA and recombinant full-length SAA indicated that serum A-SAA is a full-length SAA monomer. The photo of the membrane was shown in the lower panel.

Figure S3

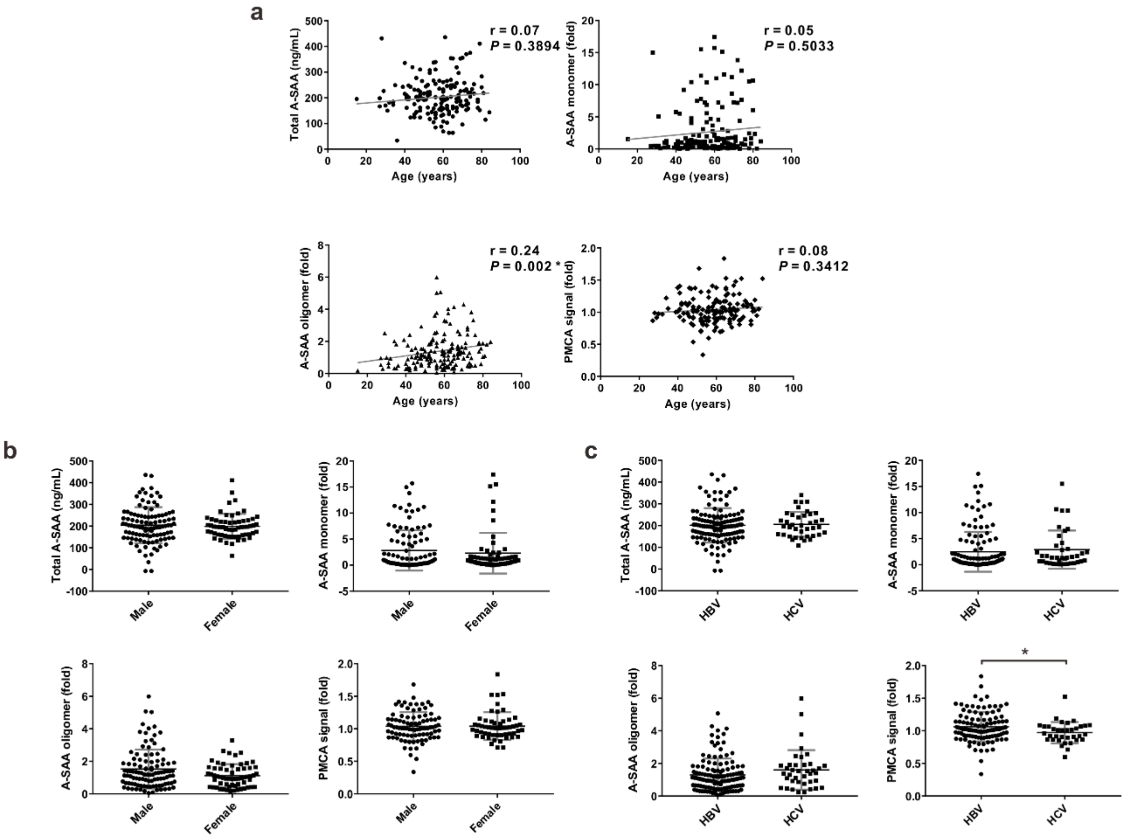

Figure S3. Association of A-SAA derived biomarkers with age, sex, and HBV/HCV infection. (**a**) Age vs. A-SAA biomarkers. The level of correlation was analyzed and evaluated by Spearman's correlation coefficient (indicated as r). (**b**) Sex vs. A-SAA biomarkers. (**c**) HBV or HCV infection vs. A-SAA biomarkers. Comparison between two groups was performed by Mann-Whitney *U* test, where $P < 0.05$ (*).

Figure S4

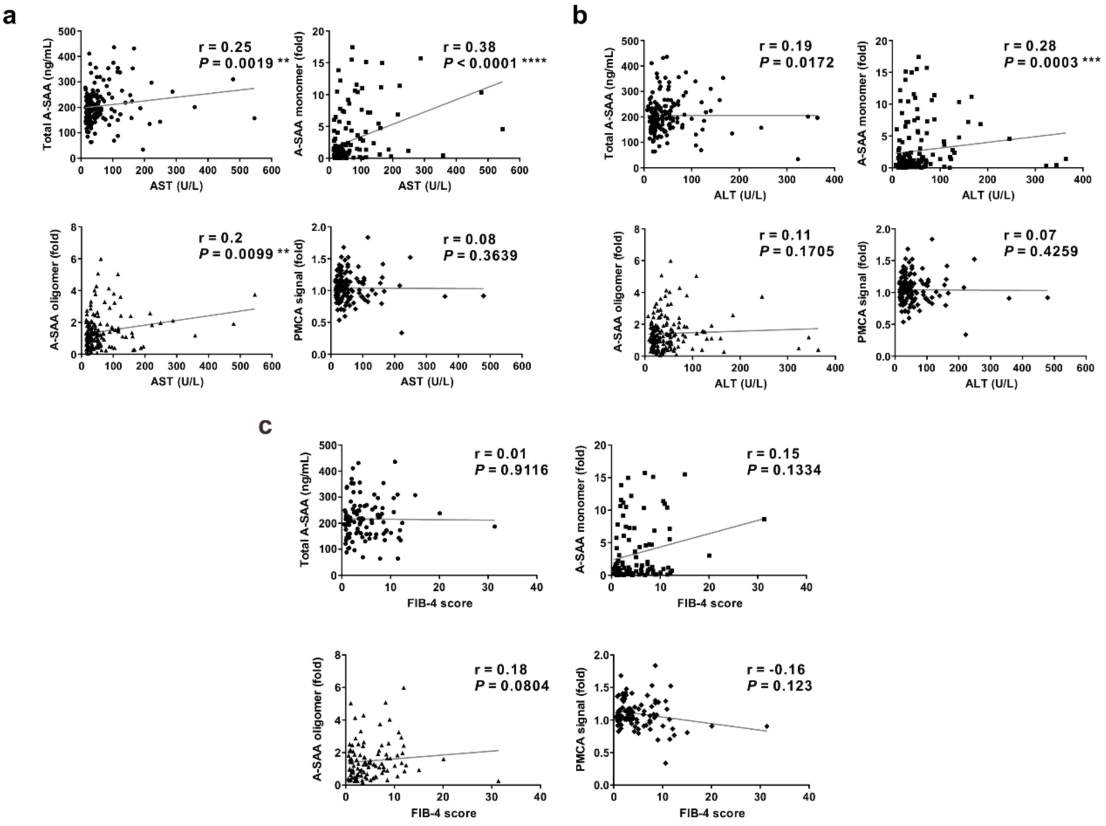

Figure S4. Association of A-SAA derived biomarkers with liver inflammation biomarkers and FIB-4 score. (**a**) AST vs. A-SAA biomarkers. (**b**) ALT vs. A-SAA biomarkers. (**c**) FIB-4 score vs. A-SAA biomarkers. The level of correlation was analyzed and evaluated by Spearman's correlation coefficient (indicated as r).

Figure S5

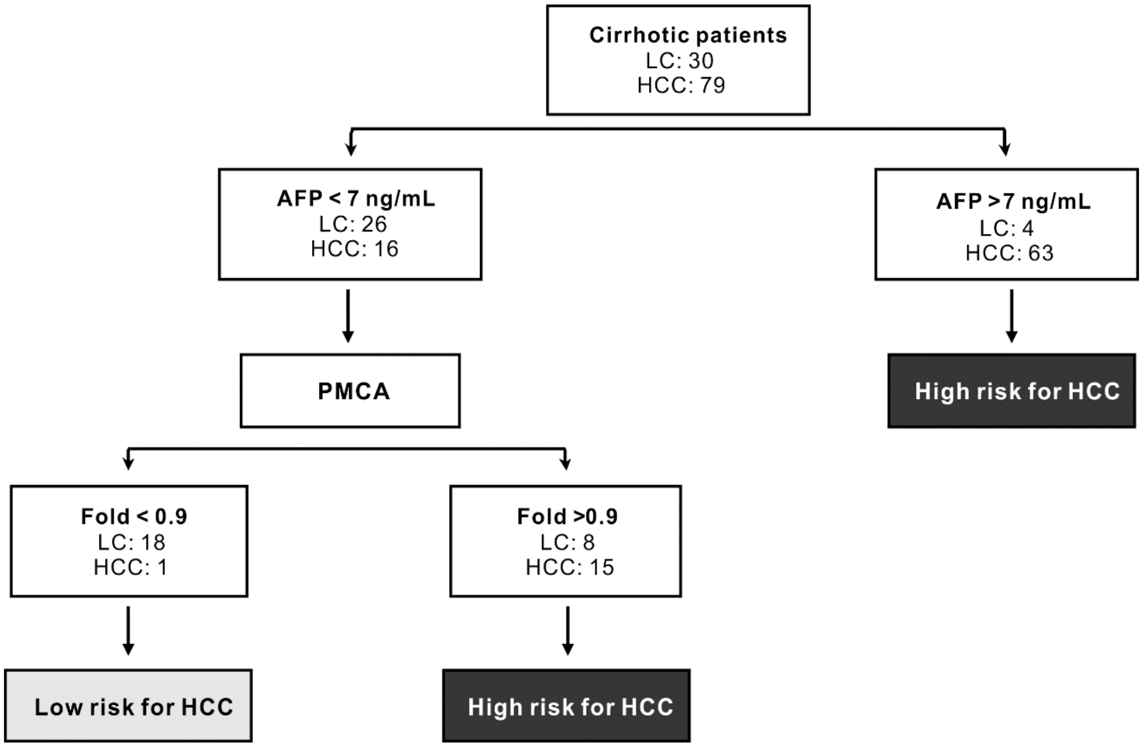

Figure S5. HCC detection scenario for cirrhotic patients by the combination of AFP and PMCA. The number of patients is shown.

Table S1. Performance of A-SAA derived biomarkers for detection of all-stage and early-stage HCC (BCLC 0-A) in all patients

		AUROC (95% CI)	*P*-value	Optimal cut-off	Sensitivity (%) (95% CI)	Specificity (%) (95% CI)	PPV (%)	NPV (%)
All-stage n = 166	Total A-SAA (ng/mL)	0.7 (0.62-0.78)	< 0.0001	200	65 (54-74)	76 (64-85)	79	61
	A-SAA monomer (fold)	0.68 (0.6-0.76)	< 0.0001	1.7	48 (38-58)	87 (77-94)	83	54
	A-SAA oligomer (fold)	0.59 (0.5-0.67)	0.0587	2.1	25 (17-35)	94 (86-98)	86	48
	PMCA signal (fold)	0.77 (0.7-0.85)	< 0.0001	1.1	58 (47-69)	92 (82-97)	91	56
	AFP (ng/mL)	0.9 (0.84-0.95)	< 0.0001	7	78 (68-86)	93 (80-98)	95	75
Early-stage n = 124	Total A-SAA (ng/mL)	0.6 (0.48-0.71)	0.0856	200	55 (40-69)	76 (65-84)	59	74
	A-SAA monomer (fold)	0.5 (0.39-0.62)	0.9904	0.04	14 (5-29)	100 (95-100)	34	0
	A-SAA oligomer (fold)	0.53 (0.42-0.65)	0.5518	0.9	52 (36-68)	63 (50-74)	33	56
	PMCA signal (fold)	0.82 (0.74-0.91)	< 0.0001	1.1	68 (53-80)	92 (82-96)	86	77
	AFP (ng/mL)	0.89 (0.81-0.96)	< 0.0001	7	74 (58-86)	93 (80-98)	89	84

Abbreviations: AFP, α-fetoprotein; A-SAA, acute-phase SAA; AUROC, area under the receiver operating characteristic curve; CI, confidence interval; HCC, hepatocellular carcinoma; NPV, negative predictive value; PMCA, protein misfolding cyclic amplification; PPV, positive predictive value.

Table S2. Performance of A-SAA derived biomarkers for detection of all-stage and early-stage HCC in cirrhotic patients with or without low AFP

		AUROC (95% CI)	*P*-value	Optimal cut-off	Sensitivity (%) (95% CI)	Specificity (%) (95% CI)	PPV (%)	NPV (%)
**All-stage** (Cirrhotic) n = 116	Total A-SAA (ng/mL)	0.75 (0.66-0.84)	< 0.0001	200	65 (54-75)	87 (69-96)	93	46
	A-SAA monomer (fold)	0.7 (0.6-0.8)	0.0011	0.4	74 (64-83)	63 (44-80)	85	44
	A-SAA oligomer (fold)	0.52 (0.41-0.64)	0.6866	1.4	45 (35-56)	70 (51-85)	81	30
	PMCA signal (fold)	0.86 (0.79-0.93)	< 0.0001	0.9	81 (71-89)	80 (61-92)	88	69
	AFP (ng/mL)	0.84 (0.76-0.92)	< 0.0001	7.3	80 (69-87)	83 (59-96)	95	60
**Early-stage** (Cirrhotic) n = 68	Total A-SAA (ng/mL)	0.65 (0.52-0.78)	0.0369	194	61 (43-76)	80 (61-92)	79	60
	A-SAA monomer (fold)	0.53 (0.39-0.67)	0.6655	0.4	58 (41-74)	63 (44-80)	66	53
	A-SAA oligomer (fold)	0.61 (0.48-0.75)	0.1057	0.9	53 (36-69)	77 (58-90)	45	27
	PMCA signal (fold)	0.91 (0.84-0.98)	< 0.0001	1	73 (56-86)	97 (83-100)	88	79
	AFP (ng/mL)	0.81 (0.7-0.93)	0.0002	7.3	74 (57-87)	83 (59-96)	88	72
**All-stage** (Cirrhotic + low AFP) n = 17	Total A-SAA (ng/mL)	0.79 (0.63-0.94)	0.0017	202	71 (44-90)	85 (65-96)	75	81
	A-SAA monomer (fold)	0.74 (0.58-0.9)	0.0079	0.4	82 (57-96)	62 (41-80)	57	80
	A-SAA oligomer (fold)	0.62 (0.44-0.81)	0.1719	0.9	53 (28-77)	85 (65-96)	29	33
	PMCA signal (fold)	0.93 (0.85-1)	< 0.0001	1	81 (54-96)	88 (70-98)	65	95
**Early-stage** (Cirrhotic + low AFP) n = 10	Total A-SAA (ng/mL)	0.67 (0.45-0.88)	0.1288	192	60 (26-88)	77 (56-91)	50	83
	A-SAA monomer (fold)	0.65 (0.44-0.86)	0.1577	0.4	80 (44-97)	62 (41-80)	41	84
	A-SAA oligomer (fold)	0.74 (0.55-0.93)	0.0285	0.9	70 (35-93)	85 (65-96)	15	40
	PMCA signal (fold)	0.94 (0.86-1)	< 0.0001	0.9	90 (56-100)	81 (61-93)	64	95

Abbreviations: AFP, α-fetoprotein; A-SAA, acute-phase SAA; AUROC, area under the receiver operating characteristic curve; CI, confidence interval; NPV, negative predictive value; PMCA, protein misfolding cyclic amplification; PPV, positive predictive value.

Table S3. Multivariable logistic regression analysis of the PMCA method to predict HCC

		Total, n (%)	Cases, n	Adjusted OR* (95% CI)
All-stage HCC	PMCA (fold)			
	<0.91 [Q1]	36 (25)	11	Reference
	0.91-1.02 [Q2]	36 (25)	18	8.43 (2.01-44.77)
	1.02-1.14 [Q3]	36 (25)	23	15 (3.86-74.36)
	≥1.14 [Q4]	36 (25)	33	101 (19.61-753.6)
Early-stage HCC	PMCA (fold)			
	<0.89 [Q1]	24 (24)	2	Reference
	0.89-1.00 [Q2]	26 (26)	8	17.43 (2.32-339.5)
	1.00-1.13 [Q3]	25 (25)	9	25.96 (3.49-512.8)
	≥1.13 [Q4]	24 (24)	21	344.8 (38.47-8286)

Abbreviations: CI, confidence interval; OR, odds ratio; PMCA, protein misfolding cyclic amplification.

*The ORs for the PMCA method were adjusted for age, gender, and AST/ALT concentration.
